# Combined impact of traditional and non-traditional health behaviors on mortality: a national prospective cohort study in Spanish older adults

**DOI:** 10.1186/1741-7015-11-47

**Published:** 2013-02-22

**Authors:** David Martínez-Gómez, Pilar Guallar-Castillón, Luz M León-Muñoz, Esther López-García, Fernando Rodríguez-Artalejo

**Affiliations:** 1 Department of Preventive Medicine and Public Health, School of Medicine, Universidad Autónoma de Madrid ⁄ IdiPAZ - CIBER of Epidemiology and Public Health (CIBERESP), Madrid, Spain

**Keywords:** aging, older adults, mortality, cohort study, smoking, physical activity, diet, sleep, sedentary, social network

## Abstract

**Background:**

Data on the combined effect of lifestyles on mortality in older people have generally been collected from highly selected populations and have been limited to traditional health behaviors. In this study, we examined the combined impact of three traditional (smoking, physical activity and diet) and three non-traditional health behaviors (sleep duration, sedentary time and social interaction) on mortality among older adults.

**Methods:**

A cohort of 3,465 individuals, representative of the Spanish population aged ≥60 years, was established in 2000/2001 and followed-up prospectively through 2011. At baseline, the following positive behaviors were self-reported: never smoking or quitting tobacco >15 years, being very or moderately physically active, having a healthy diet score ≥ median in the cohort, sleeping 7 to 8 h/d, spending <8 h/d in sitting time, and seeing friends daily. Analyses were performed with Cox regression and adjusted for the main confounders.

**Results:**

During an average nine-year follow-up, 1,244 persons died. Hazard ratios (95% confidence interval) for all-cause mortality among participants with two, three, four, five and six compared to those with zero to one positive behaviors were, respectively, 0.63 (0.46 to 0.85), 0.41 (0.31 to 0.55), 0.32 (0.24 to 0.42), 0.26 (0.20 to 0.35) and 0.20 (0.15 to 0.28) (*P *for trend <0.001). The results were similar regardless of age, sex and health status at baseline. Those with six vs. zero to one positive health behaviors had an all-cause mortality risk equivalent to being 14 years younger. Adding the three non-traditional to the four traditional behaviors improved the model fit (likelihood ratio test, *P *<0.001) and the accuracy of mortality prediction (*c*-statistic: + 0.0031, *P *= 0.040).

**Conclusions:**

Adherence to some traditional and non-traditional health behaviors may substantially reduce mortality risk in older adults.

## Background

Non-smoking, being physically active and an adequate diet have consistently been associated with improved health and reduced mortality [1]. While most studies have assessed the individual effect of each of these lifestyles, recent investigations have shown that the combined impact of these three classical or 'traditional' health behaviors is large, and may delay more than half of all deaths in the population [2-7]. Therefore, integrated action on these three factors is the cornerstone of public health strategies to reduce premature mortality [8,9]. However, there is now growing evidence that some emerging or 'non-traditional' health behaviors, such as adequate sleep duration, avoidance of excessive sedentariness and a good social network, may also prolong life [10-12].

Older adults are the fastest growing population subgroup in recent decades and make the highest use of healthcare services [13]. This has spurred research on the effect of individual health behaviors in older adults [14-19]. It is surprising, however, that to date only the HALE project has examined the combined impact of health behaviors on mortality inolder persons; moreover, the data were limited to traditional lifestyles and were obtained in highly selected populations, which limit generalization of the results [7]. Furthermore, the combined effect of some non-traditional health behaviors on mortality remains uncertain in older adults. It is also unclear whether greater adherence to the non-traditional health behaviors may improve survival beyond what is achieved by adhering to traditional healthy lifestyles.

Accordingly, this study has examined the combined impact of three traditional health behaviors (non-smoking, physical activity and adequate diet) and three non-traditional health behaviors (adequate sleep duration, no excessive sitting time and social interaction) on long-term mortality in a representative sample of the older population in Spain. This information is of high clinical and public health relevance because (i) the proportion of older adults who adhere to the traditional health behaviors is low [7] and (ii) interventions addressing multiple health behaviors might be more effective and efficient than those targeting isolated risk factors [20].

## Methods

### Study population

We used data from a cohort of 4,008 persons (1,739 men and 2,269 women) representative of the non-institutionalized population aged 60 years and older in Spain. The study methods have been reported in detail elsewhere [21,22]. Briefly, the cohort participants were recruited in 2000/2001 using probabilistic sampling by multistage clusters. The clusters were stratified according to region of residence and size of municipality. Census sections were then chosen randomly within each cluster, and the households in which information was obtained from the subjects were chosen within each section. Finally, study participants were selected in age and sex strata. Subjects, who could not participate after 10 failed visits by the interviewer, or due to disability, death, institutionalization or refusal to participate, were replaced with other individuals selected with the same sampling procedure. Baseline information was collected in the home through personal interviews and physical examination by trained and certified personnel. The study response rate was 71%.

Written informed consent was obtained from all study participants and from an accompanying family member. The study was approved by the Clinical Research Ethics Committee of the "*La Paz*" University Hospital in Madrid, Spain.

### Main exposure variables

Information on lifestyles was self-reported. Individuals reported whether they were never-, former- or current-smokers. Information was also gathered on time since quitting tobacco smoking. Physical activity was assessed with a single global question that asked participants to rate their level of physical activity as very active, moderately active, less active or inactive in comparison with their age-peers [23]. A 14-item food frequency questionnaire based on a validated instrument was administered to assess dietary patterns with the following response categories: every day, three to five days per week, one to two days per week, or never [24,25]. The consumption of fruits, vegetables, whole grain, red and processed meat, vegetable and animal fats, and fish were used to construct a healthy diet score. Participants scored +1 point for each one of the following dietary consumptions: fruits every day, vegetables every day, whole grain every day, vegetable fats every day, and fish at least three days per week. Also, participants scored -1 point for each one of the following dietary consumptions: red or processed meat every day and animal fats every day. A lower score indicates a less healthy diet.

As regards non-traditional health behaviors, sleep duration was obtained using the question (17): How many hours do you usually sleep per day (including both nighttime and daytime sleep)? Sedentary behavior was estimated by leisure time spent sitting down [26], based on the following question: How much time do you spend sitting down on weekdays? Please add up the total number of hours for all activities (eating, listening to the radio, watching television, reading, sewing, driving and so on). The same question was asked with reference to a weekend day. The number of sitting hours per week was calculated as follows: ((weekday sitting time × 5 + weekend sitting time × 2)/7). Lastly, social network was assessed as the frequency (daily, one to two times per week, one to two times per month, every few months, rarely and never) with which the individuals saw friends or neighbors.

We developed a health behavior score where each study participant was given one point for each of the following six positive behaviors: never smoker or quitting smoking >15 years, very or moderately physically active, healthy diet score ≥ median in the cohort (median = 4, healthy diet score ranging from -1 to 5), sleeping 7 to 8 h/d, sitting time <8 h/d, and daily social interaction with friends. The categorization of these health behaviors was chosen in accordance with evidence-based public health guidelines, systematic literature reviews and findings from recent prospective studies [1-19]. Thus, the health behavior score ranged from 0 to 6, whereas partial scores ranged from 0 to 3 for the traditional behaviors and the non-traditional behaviors.

### Outcome variable

The outcome was all-cause mortality from study baseline in 2000/2001 to the end of follow-up at 31 December 2011. Mortality was obtained by a computerized search of the National Death Index, which contains information on the vital status of all residents in Spain. The vital status was ascertained for 99.9% of the cohort.

### Covariates

Age, sex and educational attainment (no formal education, primary and secondary or higher) were recorded. Participants were also classified according to their occupational status (employed, unemployed, retired, househusband/housewife). Alcohol consumption was obtained with the frequency-quantity scale used in the Spanish National Health Survey [27]. First, participants rated their alcoholic beverage consumption among the following options: abstainer, former drinker, current habitual drinker or sporadic drinker. Second, those participants who indicated current drinking also reported the frequency and quantity of beer, wine and spirits consumed during the last year, to calculate total alcohol intake (g/d). To account for recall bias and to minimize potential sleep disorders, extreme sleep durations (≤3 or ≥16 hours) were registered.

Weight and height were measured using standardized procedures [21], and body mass index (BMI) was calculated as weight (kg) divided by squared height (m^2^). Waist circumference (WC) was measured with a non-elastic belt-type tape at the midpoint between the lowest rib and the iliac crest after breathing out normally. General obesity was defined as BMI ≥30 kg/m^2 ^and abdominal obesity as WC >102 cm in men and >88 cm in women [21]. Blood pressure was measured six times in the right arm at the level of the heart using standardized methods [22]. Measurements were taken with the participants in a seated position after a five-minute rest, using appropriately sized cuffs and calibrated mercury sphygmomanometers. Readings were taken at two-minute intervals, with the mean of the measurements used in the analyses. Hypertension was defined as systolic blood pressure ≥140 mm Hg, diastolic pressure ≥90 mm Hg, or under antihypertensive treatment. Participants were also asked: Has your doctor ever told you whether you have high (blood) cholesterol? If the answer was positive, they were considered to have hypercholesterolemia.

Finally, the following diseases diagnosed by a physician and reported by the study participant were also recorded: coronary heart disease, stroke, diabetes mellitus, hip fracture and cancer at any site.

### Statistical analysis

Of the 4,008 study participants, 543 were excluded because of missing information on lifestyle variables or covariates. Thus, the analyses were conducted with 3,465 individuals (1,524 men and 1,941 women).

Individuals with 0 or 1 positive health behavior were combined, because only a small proportion of participants had no positive health behaviors (0.3%). The associations of each traditional and non-traditional health behavior, as well as combinations of them, with mortality were summarized with hazard ratios (HRs) and their 95% confidence intervals (CI) obtained from Cox regression. Follow-up duration (number of days) was used as the time scale, which started at the date of enrollment in 2000/2001 and continued until date of death or 31 December 2011. Two Cox models were fitted. The first (basic) model adjusted for age, sex and educational attainment; the second model further adjusted for occupational status, alcohol intake, former drinker status, extreme sleep durations, BMI, waist circumference, systolic blood pressure, hypercholesterolemia, individual comorbidities and for other individual or combined health behaviors, as appropriate.

To express the difference in survival in persons with six versus zero to one positive health behaviors that is equivalent to the mortality associated with each one-year increase in chronological age, we divided the β coefficient for mortality in those with a score of six versus zero to one positive behaviors by the β coefficient for mortality associated with each yearly increase in age.

We also conducted a number of stratified analyses to examine whether age (<75 years, ≥75 years), sex (male, female) or educational attainment (no formal education, primary or higher education) modify the association between health behaviors and mortality. Similar stratifications were performed by health status (general and abdominal obesity, hypertension, hypercholesterolemia and morbidity).

We used two statistical methods [28] to assess the added contribution of individual and combined traditional and non-traditional health behaviors to mortality risk: first, the likelihood ratio test, a sensitive and global measure of model fit; and second, the area under the receiver operating characteristic curve (AUC or *c*-statistic), which is a measure of the predictive performance of Cox models.

We assessed the assumption of proportionality of hazards both graphically and by testing the significance of interaction terms for the health behavior score and years of follow-up. No evidence was found of departure from the proportional hazards assumption (*P *>0.1). All tests were two-sided and statistical significance was set at *P *<0.05. Analyses were performed with STATA^® ^(StataCorp LP, College Station, Texas, USA) version 11.1.

## Results

At baseline, the distribution of participants with zero to one, two, three, four, five and six positive health behaviors was 2.6%, 7.4%, 17.4%, 31.3%, 29.7%, and 11.6%, respectively. As compared with those exhibiting only zero to one, those with six positive behaviors were younger, had higher education and BMI, reported lower alcohol intake and lower frequency of extreme sleep durations, and were less likely to have serious diagnosed morbidity, but showed hypercholesterolemia more frequently (Table 1). Additional file 1 (Table S1) shows baseline characteristics of study participants according to the number of traditional and non-traditional positive health behaviors by sex.

**Table 1:** Baseline characteristics of cohort participants according to the number of traditional and non-traditional positive health behaviors.

	Number of positive health behaviors					
	0 to 1	2	3	4	5	6
*N*	91	257	604	1,084	1,029	400
Age, yr	76.5 ± 8.7	75.6 ± 8.9	73.2 ± 8.1	71.3 ± 7.7	70.7 ± 7.5	70.1 ± 7.0
Educational attainment						
No education	60.6	61.0	58.9	49.5	48.6	45.5
Primary	28.9	27.9	28.8	36.6	38.7	38.8
Secondary or higher	10.5	11.1	12.3	13.9	12.7	15.6
Occupational status						
Employed	10.2	15.1	11.9	10.8	10.4	10.1
Unemployed	0	0	0.8	0.6	0.9	0.4
Retired	88.3	82.8	85.8	85.2	86.6	88.2
Househusband/housewife	1.5	2.1	1.6	3.5	2.1	1.3
Alcohol intake, g/d	26.8 ± 71.9	17.8 ± 36.2	19.0 ± 43.6	15.3 ± 40.0	11.3 ± 28.5	9.8 ± 23.9
Former drinking	24.5	23.6	13.6	12.7	7.4	9.7
Extreme sleep durations ^a^	5.5	3.2	2.8	2.1	1.4	0
Body mass index, kg/m^2^	28.1 ± 5.1	28.6 ± 5.2	28.9 ± 4.9	28.9 ± 4.2	28.9 ± 4.5	28.8 ± 4.0
Waist circumference, cm	98.9 ± 11.8	99.6 ± 12.7	100.2 ± 14.0	99.2 ± 11.3	97.9 ± 11.3	98.0 ± 11.6
Systolic blood pressure, mm Hg	145.4 ± 21.7	145.5 ± 21.9	144.7 ± 20.4	142.8 ± 18.7	142.1 ± 18.6	143.4 ± 18.5
Hypercholesterolemia	12.6	25.6	24.8	25.0	25.9	27.1
Comorbidities						
Coronary heart disease	10.5	8.7	4.1	2.8	1.2	1.8
Stroke	9.6	9.0	8.2	4.9	5.0	3.9
Diabetes mellitus	19.0	17.9	15.5	14.3	14.4	13.3
Hip fracture	8.3	4.6	2.8	2.5	2.3	0.4
Cancer	1.6	3.0	2.9	1.4	1.6	1.5

Values are mean ± SD or %. Participants scored one point for each health behavior: never smoking or quitting tobacco >15 years; being very/moderately physically active; healthy diet score ≥median in the cohort; sleeping 7 to 8 h/d; sitting time <8 h/d; interaction with friends daily. ^a ^Sleeping ≤3 or ≥16 h/day.

During an average follow-up of 9.0 years and 31,185 person-years of observation, a total of 1,244 deaths (35.9%) occurred. Table 2 shows the association of each traditional and non-traditional lifestyle with mortality. In the basic model, each health behavior was associated with lower mortality. After additional adjustment for all potential confounders and the other health behaviors, the associations were slightly attenuated but remained statistically significant, with the exception of seeing friends daily, which showed a marginal association with reduced mortality (HR = 0.92, 95% CI: 0.79 to 1.08). Among the traditional health behaviors, being very or moderately physically active showed the greatest impact on reduced mortality (HR = 0.63, 95% CI: 0.55 to 0.72). Among the non-traditional health behaviors, avoiding excessive sitting had the strongest inverse association (HR = 0.70, 95% CI: 0.60 to 0.82). Given that physical activity and sitting time represent different components of the energy expenditure continuum, we also assessed the association between a global measure of activity and mortality. As compared with individuals who were less active/inactive and spent ≥8 h/d seated, those who were very/moderately physically active and spent <8 h/d seated showed a fully-adjusted mortality HR = 0.44 (95% CI: 0.36 to 0.52).

**Table 2:** Mortality risk according to traditional and non-traditional health behaviors in older adults

	*N */deaths	Model 1 HR (95% CI)	Model 2 HR (95% CI)
**Traditional health behavior**			
Never smoking or quitting tobacco >15 years			
No	793/331	1 (Ref.)	1 (Ref.)
Yes	2,672/913	0.61 (0.51 to 0.73)	0.65 (0.55 to 0.78)
Very/moderately physically active			
No	855/478	1 (Ref.)	1 (Ref.)
Yes	2,610/766	0.55 (0.48 to 0.63)	0.63 (0.55 to 0.72)
Healthy diet score ≥median in the cohort			
No	1,741/695	1 (Ref.)	1 (Ref.)
Yes	1,724/549	0.80 (0.70 to 0.90)	0.79 (0.70 to 0.89)
			
**Non-traditional health behavior**			
Sleeping 7 to 8 h/d			
No	2,062/840	1 (Ref.)	1 (Ref.)
Yes	1,403/404	0.76 (0.67 to 0.87)	0.83 (0.73 to 0.94)
Sitting time <8 h/d			
No	479/288	1 (Ref.)	1 (Ref.)
Yes	2,986/956	0.56 (0.47 to 0.65)	0.70 (0.60 to 0.82)
Interaction with friends daily			
No	573/269	1 (Ref.)	1 (Ref.)
Yes	2,892/975	0.78 (0.65 to 0.93)	0.92 (0.79 to 1.08)

HR, Hazard ratio; CI, Confidence interval. Model 1: Adjusted for age (years), sex (men, women), and educational attainment (no education, primary, secondary or higher). Model 2: Adjusted as in Model 1 and additionally for occupational status (employed, unemployed, retired, househusband/housewife), alcohol intake (g/d), former drinking (yes, no), extreme sleep durations (yes, no), body mass index (kg/m^2^), waist circumference (cm), systolic blood pressure (mm Hg), hypercholesterolemia status (yes, no), coronary heart disease (yes, no), stroke (yes, no), diabetes mellitus (yes, no), hip fracture (yes, no), cancer (yes, no), and the other health behaviors included in this table.

**Figure 1:**
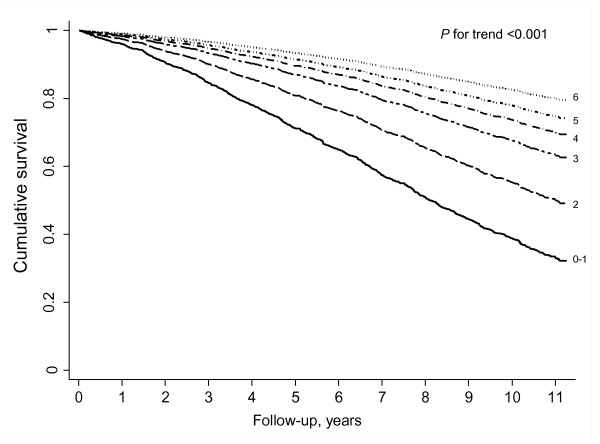
**Survival function according to number of traditional and non-traditional health behaviors in Spanish older adults**. Survival function adjusted for age (years), sex (men, women), educational attainment (no education, primary, secondary or higher), body mass index (kg/m^2^), waist circumference (cm), systolic blood pressure (mm Hg), hypercholesterolemia status (yes, no), alcohol intake (g/d), former drinker (yes, no), extreme sleep durations (yes, no), occupational status (employed, unemployed, retired, househusband/housewife), and history of coronary heart disease (yes, no), stroke (yes, no), diabetes mellitus (yes, no), hip fracture (yes, no), and cancer (yes, no).

Table 3 shows the mortality risk according to the number of positive health behaviors. An increasing number of traditional behaviors was associated with progressively lower mortality (*P *for trend <0.001), even after adjustment for all potential confounders and the non-traditional health behaviors. Likewise, the number of non-traditional behaviors showed an independent association with reduced mortality (*P *for trend <0.001) independently of potential confounders and the traditional health behaviors. As a result, the higher the number of positive behaviors (traditional plus non-traditional), the lower the mortality risk (*P *for trend <0.001). In the fully-adjusted analyses, the HR (95% CI) for all-cause mortality among participants with six positive behaviors compared to those with one or none was 0.20 (0.15 to 0.28). Figure 1 shows that the mortality reduction associated with healthy lifestyles is observed early, in particular, from the second year of follow-up. The reduced mortality risk in those with six versus zero to one health behaviors was equivalent to a reduction of 14 years in chronological age: in the fully adjusted regression model, the β coefficients were -1.60 for six health behaviors compared to those who scored zero to one, and +0.11 for each subsequent yearly increase in chronological age.

**Table 3:** Mortality risk according to number of traditional and non-traditional positive health behaviors in Spanish older adults

	Number of positive health behaviors						
		
	0 to 1	2	3	4	5	6	*P *_for trend_
**3 traditional health behaviors ^a^**							
*N */ deaths	871/464	1,564/518	1,030/262				
Model 1- HR (95% CI)	1 (Ref.)	0.56 (0.48 to 0.65)	0.45 (0.38 to 0.53)	NA	NA	NA	<0.001
Model 2- HR (95% CI)	1 (Ref.)	0.61 (0.52 to 0.71)	0.51 (0.43 to 0.60)	NA	NA	NA	<0.001
**3 non-traditional health behaviors ^b^**							
*N */ deaths	615/345	1,767/613	1,083/286				
Model 1- HR (95% CI)	1 (Ref.)	0.68 (0.58 to 0.80)	0.54 (0.45 to 0.65)	NA	NA	NA	<0.001
Model 2- HR (95% CI)	1 (Ref.)	0.77 (0.66 to 0.90)	0.64 (0.53 to 0.76)	NA	NA	NA	<0.001
**3 traditional + 3 non-traditional health behaviors**							
*N */ deaths	91/73	257/167	604/285	1,084/367	1,029/267	400/85	
Model 1- HR (95% CI)	1 (Ref.)	0.62 (0.46 to 0.83)	0.40 (0.30 to 0.53)	0.31 (0.24 to 0.41)	0.25 (0.19 to 0.33)	0.20 (0.14 to 0.27)	<0.001
Model 2- HR (95% CI)	1 (Ref.)	0.63 (0.46 to 0.85)	0.41 (0.31 to 0.55)	0.32 (0.24 to 0.42)	0.26 (0.20 to 0.35)	0.20 (0.15 to 0.28)	<0.001

CI, Confidence interval; HR, Hazard ratio. ^a ^Traditional health behaviors: never smoking or quitting tobacco >15 years; being very/moderately physically active; having a healthy diet score ≥ median in the cohort. ^b ^Non-traditional health behaviors: sleeping 7 to 8 h/d; sitting time <8 h/d; interaction with friends daily. Models 1 and 2 are adjusted as in Table 2.

To rule out preexisting disease (for example, cardiovascular disease or cancer) as a factor leading to changes in lifestyle and its association with mortality, we repeated the analysis after excluding the 196 deaths occurring in the first two years of follow-up. The resulting mortality HR (95% CI) for those with six versus zero to one positive health behaviors was virtually the same: 0.21 (0.14 to 0.30) (*P *for trend <0.001).

Table 4 shows the added contribution of individual and combined positive health behaviors to prediction of mortality risk in the fully adjusted Cox model. According to the likelihood ratio test, all individual and combined health behaviors improved model fit (all *P *≤0.001). The *c*-statistic also indicates that each individual behavior, with the exception of diet (*P *= 0.101) and seeing friends daily (*P *= 0.073), as well as all combinations of health behaviors improve risk prediction. Moreover, both statistical methods suggest that prediction of mortality risk based on the combination of traditional and non-traditional health behaviors is better than the prediction based on traditional behaviors alone (*χ^2 ^*= 26.09*, P *<0.001; *c*-statistic = + 0.0031, *P *= 0.040).

**Table 4:** Contribution of individual and combined traditional and non-traditional health behaviors to mortality prediction in Spanish older adults

	***χ*^2^**	***P***	***c-*statistic**	***P***
**Fully adjusted model **	...	...	0.7525	...
+ Smoking	28.82	<0.001	0.7562	0.002
+ Physical activity	69.57	<0.001	0.7614	<0.001
+ Diet	16.66	<0.001	0.7540	0.101
+ Smoking + physical activity	98.76	<0.001	0.7645	<0.001
+ Smoking + diet	44.37	<0.001	0.7574	0.001
+ Physical activity + diet	86.31	<0.001	0.7624	<0.001
+ 3 traditional behaviors	94.84	<0.001	0.7627	<0.001
+ Sleeping	20.34	<0.001	0.7548	0.021
+ Sitting time	39.56	<0.001	0.7562	0.003
+ Social interaction	10.41	0.001	0.7537	0.073
+ Sleeping + sitting time	56.07	<0.001	0.7581	<0.001
+ Sleeping + social interaction	29.90	<0.001	0.7559	0.004
+ Sitting time + social interaction	46.82	<0.001	0.7569	0.001
+ 3 non-traditional behaviors	48.53	<0.001	0.7576	<0.001
+ 3 traditional behaviors + sleeping	110.19	<0.001	0.7644	<0.001
+ 3 traditional behaviors + sitting time	118.35	<0.001	0.7644	<0.001
+ 3 traditional behaviors + social interaction	97.57	<0.001	0.7630	<0.001
+ 3 traditional behaviors + sleeping + sitting time	131.84	<0.001	0.7659	<0.001
+ 3 traditional behaviors + sleeping + social interaction	112.66	<0.001	0.7647	<0.001
+ 3 traditional behaviors + sitting time + social interaction	120.33	<0.001	0.7645	<0.001
+ 3 non-traditional behaviors + smoking	73.77	<0.001	0.7606	<0.001
+ 3 non-traditional behaviors + physical activity	94.78	<0.001	0.7633	<0.001
+ 3 non-traditional behaviors + diet	61.79	<0.001	0.7587	<0.001
+ 3 non-traditional behaviors + smoking + physical activity	121.06	<0.001	0.7662	<0.001
+ 3 non-traditional behaviors + smoking + diet	86.26	<0.001	0.7615	<0.001
+ 3 non-traditional behaviors + physical activity + diet	108.86	<0.001	0.7643	<0.001
+ 3 traditional + 3 non-traditional behaviors ^a^	134.01 ^a^	<0.001	0.7658 ^b^	<0.001

The fully adjusted model includes age (years), sex (men, women), educational attainment (no education, primary, secondary or higher), occupational status (employed, unemployed, retired, househusband/housewife), alcohol intake (g/d), former drinking (yes, no), extreme sleep duration (yes, no), body mass index (kg/m^2^), waist circumference (cm), systolic blood pressure (mm Hg), hypercholesterolemia status (yes, no), and history of coronary heart disease (yes, no), stroke (yes, no), diabetes mellitus (yes, no), hip fracture (yes, no), and cancer (yes, no).+ indicates the addition of each variable or variables to the full model. *χ*^2 ^is the likelihood ratio statistic for each variable or variables when added to the full model. *P-*values for pairwise comparisons between the presented model and the full model. Traditional behaviors: smoking, physical activity and diet. Non-traditional behaviors: sleeping, sitting time and social interaction with friends. ^a ^*P *<0.001 compared with the model with three traditional behaviors combined. ^b ^*P *= 0.040 compared with the model with three traditional behaviors combined.

Additional files 2 and 3 (Tables S2 and S3) show that the inverse dose-response relation between the number of positive health behaviors and mortality held regardless of socio-demographic group, BMI, WC, hypertension, hypercholesterolemia or diagnosed morbidity. Of particular interest is that the association was observed even in those with one or more diagnosed severe diseases, which again suggests that pre-existing subclinical disease is unlikely to explain the overall study results.

## Discussion

In this cohort of Spanish older adults, the number of positive health behaviors showed an inverse dose-response relation with mortality risk. Older adults with six positive behaviors had an 80% lower mortality risk or the equivalent to a reduction of 14 years in chronological age compared to those with zero to one health behaviors. Importantly, this survival benefit was not completely due to the traditional healthy lifestyles (not smoking, being physically active and adequate diet). In fact, some non-traditional health behaviors, such as adequate sleep duration, avoiding excessive sitting time, and social interaction with friends, were important contributors to improved survival.

Age and sex are the most important predictors of mortality [29]. These and other factors (for example, genetics) cannot, however, be modified. Smoking, physical activity and diet are the three potentially modifiable lifestyles with strongest evidence of their role in mortality [1]. There is also evidence that each health behavior "counts" and that the higher the number of these three health behaviors, the lower the mortality risk in adults [2-7]. Our findings extend previous observations limited to traditional health behaviors, suggesting that health promotion interventions in older adults should also focus on non-traditional healthy lifestyles to obtain full benefit.

A recent systematic review and meta-analysis of prospective studies indicates that both short and long duration of sleep are significant predictors of death [10]. In older adults, long sleep may even be more detrimental on mortality than short sleep [17]. Although the science of sedentary behavior is relatively new, most studies have shown a dose-response association between sitting time and mortality, which is independent of physical activity [11]. Moreover, a poor social network has also been consistently linked to higher mortality [12], especially in older people [19].

In the present study, the three non-traditional health behaviors showed protective associations with mortality. In fact, the impact of sitting time on mortality was close to that exerted by smoking, which is the leading preventable cause of death. The combined impact of the number of non-traditional health behaviors was also substantial, as seen in the fact that older adults with these three healthy lifestyles had a 36% lower mortality than those with one or none. Likewise, both liberal (likelihood ratio test) and conservative (*c*-statistic) statistical methods have indicated that adding these three new lifestyles to the three classical health behaviors may improve mortality prediction. Taken together, these findings support the assertion that in older adults some non-traditional lifestyles may increase longevity beyond what is achieved by the three classical health behaviors.

To our knowledge, only the HALE project has examined the combined effect of health behaviors on mortality in older persons [7]. In this study, Knoops *et al*. investigated the combined effect of four health behaviors (healthy diet, being physically active, moderate alcohol use and non-smoking) on 10-year mortality among 2,339 individuals aged 70 to 90 years from 11 European countries. Adherence to these four health behaviors was associated with lower mortality compared with those who adhered to only one or no health behaviors. As some researchers have pointed out [3,30,31], the HALE project included a highly selected group of older adults (for example, participants had a high educational level, only 36% were women and over 50% of the women drank alcohol regularly), and the consistency of findings was not examined within subpopulations defined by demographic (for example, age and sex) or health conditions (for example, obesity). Also of note is that lifestyles (for example, physical activity) were measured with different methods across the study populations. Our study, while overcoming these limitations, confirmed the results of the HALE project. One further advantage of our study is that the results correspond to a single, relatively homogeneous population showing the usual range of lifestyle variations that may be more realistically achievable and directly relevant to immediate public health [3]. Moreover, in contrast to some complex instruments used in HALE to assess diet or physical activity, our lifestyle score was based on very simple and easy to ask questions, which may be of practical use in clinical practice or for monitoring health in population surveys.

Other relevant findings in our study were the consistency of results across subgroups defined by socio-demographic variables and health conditions, and the improved survival associated with positive health behaviors that was observed since the second year of follow-up. These results suggest that it is never too late - even at age 75 or older - to adopt a healthy lifestyle, and that most older adults (whether or not they have severe disease) may benefit, although further testing in experimental studies is needed to support this contention.

This study has several strengths. It included a representative sample of older adults in Spain, which allows for generalization of results. In fact, in our cohort the annual mortality rates are only slightly lower than in the Spanish population of the same age and sex. This was to be expected because our cohort exclusively included non-institutionalized individuals and participants in epidemiological studies tend to be healthier than in the general population. Likewise, all study variables were obtained by well-trained interviewers using standardized methods. The relatively large sample size made it possible to examine whether the study associations varied according to socio-demographic characteristics and many health conditions. Moreover, analyses were adjusted for a substantial number of confounders. Lastly, to reduce reporting bias linked to disease status, the analyses have been replicated in individuals without severe comorbidity, and also after excluding deaths in the first two years of follow-up.

Our study also had some limitations. First, information on lifestyles was obtained at baseline. Although our analyses assume that lifestyles have certain stability over time, some changes are still possible and would likely have led to an underestimation of the protective impact of health behaviors on mortality. Second, lifestyle was self-reported, which may have led to recall bias, particularly for assessing physical activity and non-traditional health behaviors. However, similar measures of physical activity, sleep duration and sitting time have shown adequate validity compared with objective measures in adults and older adults [23,32-34]. Third, health behaviors related to physical activity and diet could not be defined according to public health recommendations (for example, 150 minutes/week for physical activity, and at least two servings/day of fruit and vegetables). Moreover, health behaviors were dichotomized; if people who had a high-risk behavior falsely reported having a healthy behavior, it is likely that the hazard ratios may have been underestimated. Also misclassification error may differ between health behaviors, so that our results on the relative influence of each behavior on mortality should be interpreted with caution. Therefore, taken together, these limitations in the measurement of health behaviors have precluded a better understanding of the study associations.

Future research should assess the combined effect of other non-traditional health behaviors (for example, participating in a dancing group, playing a musical instrument, religious activities and so on) on mortality in older adults. It should also examine whether multiple healthy lifestyles might help to maintain a long and disease-free independent life and a good health-related quality of life.

## Conclusions

The combination of six healthy behaviors was associated with a substantial reduction of mortality in older adults, regardless of age, sex, educational attainment and many health conditions. Among these lifestyles, some non-traditional health behaviors, such as sleep duration, sitting time and social interaction, seem to contribute importantly to improved survival. These results are of particular relevance for countries like Spain, where life expectancy in the population is very long (both at birth and at age 60) [35], because they suggest that longevity can be further increased when older adults adopt a healthy lifestyle.

## Abbreviations

BMI: body mass index; HALE: Healthy Ageing: a Longitudinal study in Europe; WC: waist circumference

## Competing interests

The authors declare that they have no competing interests.

## Authors' contributions

DMG and FRA had full access to all of the data in the study and take responsibility for the integrity of the data and the accuracy of the data analysis. DMG, PGC, ELG and FRA contributed to the study concept and design. PGC, LLM and ELG acquired the data. DMG, PGC and FRA analyzed and interpreted the data.: DMG and FRA drafted the manuscript. DMG, PGC, LLM, ELG and FRA critically revised the manuscript for important intellectual content. DMG, PGC and FRA performed the statistical analysis. PGC and FRA obtained the funding. PGC, LLM and ELG provided the administrative, technical or material support. FRA supervised the study. All authors have read and approved the final manuscript.

## Pre-publication history

The pre-publication history for this paper can be accessed here:

http://www.biomedcentral.com/1741-7015/11/47/prepub

## Supplementary Material

Additional file 1 **Table S1**. Baseline characteristics of cohort participants according to the number of traditional and non-traditional positive health behaviors in Spanish older adults, by sex Click here for file

Additional file 2 **Table S2**. Mortality risk according to number of traditional and non-traditional positive health behaviors in Spanish older adults stratified by age, sex, and educational attainment Click here for file

Additional file 3 **Table S3**. Mortality risk according to number of traditional and non-traditional positive health behaviors in Spanish older adults, stratified by health conditions. Click here for file
