# Supplementary material for: Combined impact of traditional and non-traditional health behaviors on mortality: a national prospective cohort study in Spanish older adults
Source: BMC Med. 2013 Feb 22;11:47. doi: 10.1186/1741-7015-11-47 (PMC3621845; doi:10.1186/1741-7015-11-47)
Supplement: Additional file 1 — Table S1. Baseline characteristics of cohort participants according to the number of traditional and non-traditional positive health behaviors in Spanish older adults, by sex [file 1741-7015-11-47-S1.DOC]

|  | Number of positive health behaviors | | | | | |
| --- | --- | --- | --- | --- | --- | --- |
|  | 0-1 | 2 | 3 | 4 | 5 | 6 |
| **Men***, N* | 55 | 114 | 329 | 513 | 376 | 137 |
| Age, yr | 74.7±8.6 | 73.1±8.7 | 71.8±7.6 | 70.6±7.3 | 70.1±6.8 | 70.7±6.7 |
| Educational level |  |  |  |  |  |  |
| No education | 52.6 | 48.3 | 51.3 | 45.8 | 39.8 | 38.4 |
| Primary | 36.1 | 35.1 | 32.9 | 34.8 | 41.4 | 34.9 |
| Secondary or higher | 11.3 | 16.6 | 15.8 | 19.4 | 18.8 | 26.7 |
| Occupational status |  |  |  |  |  |  |
| Employed | 5.5 | 11.1 | 11.0 | 11.2 | 9.3 | 9.7 |
| Unemployed | 0 | 0 | 1.1 | 0.3 | 1.5 | 0 |
| Retired | 94.5 | 88.9 | 87.9 | 87.9 | 89.1 | 90.3 |
| Househusband | 0 | 0 | 0 | 0.6 | 0 | 0 |
| Alcohol intake, g/d | 41.5±89.8 | 35.5±48.2 | 32.6±54.4 | 28.4±54.0 | 24.0±41.2 | 22.9±35.6 |
| Former drinker | 31.2 | 38.8 | 20.1 | 18.9 | 12.2 | 19.6 |
| Extreme sleep durations ^a^ | 1.5 | 2.3 | 2.3 | 1.6 | 0.3 | 0 |
| Body mass index, kg/m^2^ | 27.2±4.5 | 27.2±3.8 | 28.2±4.3 | 28.5±3.5 | 28.2±3.9 | 28.0±3.1 |
| Waist circumference, cm | 100.5±11.2 | 101.9±11.3 | 101.9±14.4 | 102.3±9.4 | 101.2±10.7 | 101.0±9.1 |
| Systolic blood pressure, mm Hg | 146.9±21.2 | 145.0±21.2 | 144.9±20.2 | 141.2±17.3 | 141.8±18.3 | 142.4±18.9 |
| Hypercholesterolemia | 12.0 | 26.3 | 23.0 | 21.6 | 21.8 | 20.3 |
| Comorbidities |  |  |  |  |  |  |
| Coronary heart disease | 7.2 | 11.1 | 3.5 | 1.9 | 0.5 | 0.4 |
| Stroke | 8.3 | 13.6 | 9.7 | 6.4 | 7.6 | 6.8 |
| Diabetes mellitus | 17.1 | 17.9 | 12.2 | 14.1 | 13.6 | 10.2 |
| Hip fracture | 0 | 0 | 2.7 | 2.0 | 2.8 | 0.6 |
| Cancer | 2.7 | 4.3 | 2.2 | 2.0 | 0.6 | 0.6 |
|  |  |  |  |  |  |  |
| **Women***, N* | 36 | 143 | 275 | 571 | 653 | 263 |
| Age, yr | 79.1±8.2 | 77.6±8.5 | 74.7±8.3 | 71.9±8.0 | 71.0±7.8 | 69.9±7.2 |
| Educational level |  |  |  |  |  |  |
| No education | 72.6 | 71.0 | 68.0 | 52.7 | 53.7 | 49.2 |
| Primary | 18.1 | 22.2 | 24.0 | 38.2 | 37.2 | 40.9 |
| Secondary or higher | 9.3 | 6.8 | 8.0 | 9.1 | 9.1 | 9.9 |
| Occupational status |  |  |  |  |  |  |
| Employed | 17.2 | 18.3 | 12.9 | 10.5 | 11.0 | 10.4 |
| Unemployed | 0 | 0 | 0.4 | 0.8 | 0.5 | 0.7 |
| Retired | 78.9 | 78.0 | 83.3 | 82.8 | 85.1 | 87.1 |
| Housewife | 3.9 | 3.8 | 3.4 | 6.0 | 3.4 | 1.9 |
| Alcohol intake, g/d | 4.6±8.9 | 3.8±8.2 | 2.8±13.0 | 3.7±10.8 | 3.9±12.3 | 3.0±8.4 |
| Former drinker | 14.4 | 11.4 | 5.8 | 7.1 | 4.6 | 4.6 |
| Extreme sleep durations ^a^ | 11.5 | 3.9 | 3.4 | 2.6 | 2.1 | 0 |
| Body mass index, kg/m^2^ | 29.6±5.6 | 29.7±5.9 | 29.8±5.4 | 29.3±4.8 | 29.3±4.8 | 29.3±4.3 |
| Waist circumference, cm | 96.5±12.3 | 97.7±13.4 | 98.2±13.2 | 96.4±12.1 | 96.0±11.2 | 96.4±12.4 |
| Systolic blood pressure, mm Hg | 143.2±22.4 | 146.0±22.6 | 144.5±20.8 | 144.2±19.8 | 142.2±18.9 | 143.9±18.2 |
| Hypercholesterolemia | 13.5 | 25.1 | 27.0 | 28.0 | 28.2 | 30.6 |
| Comorbidities |  |  |  |  |  |  |
| Coronary heart disease | 15.3 | 6.8 | 5.0 | 3.5 | 1.6 | 2.5 |
| Stroke | 11.6 | 5.3 | 6.4 | 3.5 | 3.5 | 2.4 |
| Diabetes mellitus | 21.9 | 17.9 | 19.5 | 14.5 | 14.9 | 15.0 |
| Hip fracture | 20.9 | 8.3 | 2.9 | 3.0 | 2.0 | 0.3 |
| Cancer | 0 | 1.9 | 3.9 | 0.9 | 2.2 | 1.9 |

Table S1. Baseline characteristics of cohort participants according to the number of traditional and non-traditional positive health behaviors in Spanish older adults, by sex.

Values are mean ± SD or %. Participants scored one point for each health behavior: never smoking or quitting tobacco >15 years; being very/moderately physically active; healthy diet score ≥ median in the cohort; sleeping 7-8 h/d; sitting time <8 h/d; interaction with friends daily. ^a^ Sleeping ≤3 or ≥16 h/day.
