# Supplementary material for: Combined impact of traditional and non-traditional health behaviors on mortality: a national prospective cohort study in Spanish older adults
Source: BMC Med. 2013 Feb 22;11:47. doi: 10.1186/1741-7015-11-47 (PMC3621845; doi:10.1186/1741-7015-11-47)
Supplement: Additional file 2 — Table S2. Mortality risk according to number of traditional and non-traditional positive health behaviors in Spanish older adults stratified by age, sex, and educational attainment [file 1741-7015-11-47-S2.DOC]

Table S2. Mortality risk according to number of traditional and non-traditional positive health behaviors in Spanish older adults stratified by age, sex, and educational attainment

|  | Number of positive health behaviors | | | | | |  |
| --- | --- | --- | --- | --- | --- | --- | --- |
|  | 0-1 | 2 | 3 | 4 | 5 | 6 | *P* _for trend_ |
| **<75 years** |  |  |  |  |  |  |  |
| *N* / deaths | 43/29 | 118/45 | 353/103 | 762/168 | 719/102 | 305/38 |  |
| Adjusted HR (95%CI)^a^ | 1 (Ref.) | 0.64 (0.39-1.07) | 0.47 (0.31-0.70) | 0.34 (0.23-0.51) | 0.23 (0.15-0.35) | 0.19 (0.12-0.31) | <0.001 |
| **≥75 years** |  |  |  |  |  |  |  |
| *N* / deaths | 47/44 | 139/122 | 252/182 | 322/199 | 309/165 | 96/47 |  |
| Adjusted HR (95%CI) ^a^ | 1 (Ref.) | 0.55 (0.37-0.80) | 0.33 (0.23-0.47) | 0.24 (0.17-0.34) | 0.20 (0.14-0.30) | 0.16 (0.10-0.26) | <0.001 |
| **Men** |  |  |  |  |  |  |  |
| *N* / deaths | 55/46 | 114/76 | 329/152 | 513/184 | 376/113 | 137/36 |  |
| Adjusted HR (95%CI) ^a^ | 1 (Ref.) | 0.64 (0.41-1.00) | 0.38 (0.25-0.57) | 0.31 (0.21-0.45) | 0.26 (0.17-0.40) | 0.19 (0.12-0.31) | <0.001 |
| **Women** |  |  |  |  |  |  |  |
| *N* / deaths | 36/27 | 143/90 | 275/134 | 571/183 | 653/154 | 263/49 |  |
| Adjusted HR (95%CI) ^a^ | 1 (Ref.) | 0.66 (0.44-0.99) | 0.50 (0.34-0.74) | 0.38 (0.26-0.55) | 0.29 (0.20-0.43) | 0.24 (0.15-0.38) | <0.001 |
| **No formal education** |  |  |  |  |  |  |  |
| *N* / deaths | 55/47 | 157/107 | 356/172 | 536/188 | 500/141 | 182/39 |  |
| Adjusted HR (95%CI) | 1 (Ref.) | 0.63 (0.44-0.92) | 0.40 (0.28-0.57) | 0.31 (0.22-0.45) | 0.25 (0.17-0.36) | 0.18 (0.11-0.29) | <0.001 |
| **Primary or higher education** |  |  |  |  |  |  |  |
| *N* / deaths | 36/26 | 100/60 | 248/113 | 548/179 | 529/126 | 218/46 |  |
| Adjusted HR (95%CI) ^a^ | 1 (Ref.) | 0.58 (0.35-0.95) | 0.40 (0.26-0.62) | 0.31 (0.21-0.48) | 0.26 (0.17-0.41) | 0.21 (0.13-0.34) | <0.001 |

HR: Hazard ratio; CI: Confidence interval. ^a^HRs are adjusted as in Model 2 in table 2. Positive health behaviors: never smoking or quitting tobacco >15 years; being very/

moderately physically active; having a healthy diet score ≥median in the cohort; sleeping 7-8 h/d; sitting time <8 h/d; interaction with friends daily.
