# Supplementary material for: Combined impact of traditional and non-traditional health behaviors on mortality: a national prospective cohort study in Spanish older adults
Source: BMC Med. 2013 Feb 22;11:47. doi: 10.1186/1741-7015-11-47 (PMC3621845; doi:10.1186/1741-7015-11-47)
Supplement: Additional file 3 — Table S3. Mortality risk according to number of traditional and non-traditional positive health behaviors in Spanish older adults, stratified by health conditions. [file 1741-7015-11-47-S3.DOC]

Table S3. Mortality risk according to number of traditional and non-traditional positive health behaviors in Spanish older adults, stratified by health conditions

|  | Number of positive health behaviors | | | | | |  |
| --- | --- | --- | --- | --- | --- | --- | --- |
|  | 0-1 | 2 | 3 | 4 | 5 | 6 | *P* _for trend_ |
| **<30 kg/m^2^ body mass index** |  |  |  |  |  |  |  |
| *N* / deaths | 66/56 | 174/122 | 400/201 | 680/246 | 659/183 | 268/58 |  |
| Adjusted HR (95%CI)^a^ | 1 (Ref.) | 0.61 (0.43-0.87) | 0.42 (0.30-0.58) | 0.32 (0.24-0.44) | 0.26 (0.19-0.36) | 0.19 (0.13-0.28) | <0.001 |
| **≥30 kg/m^2^ body mass index** |  |  |  |  |  |  |  |
| *N* / deaths | 25/17 | 83/45 | 205/84 | 404/121 | 369/84 | 132/27 |  |
| Adjusted HR (95%CI) ^a^ | 1 (Ref) | 0.71 (0.38-1.32) | 0.44 (0.24-0.83) | 0.37 (0.20-0.67) | 0.30 (0.16-0.56) | 0.28 (0.14-0.56) | <0.001 |
| **No abdominal obesity** |  |  |  |  |  |  |  |
| *N* / deaths | 31/28 | 88/62 | 244/113 | 388/131 | 346/94 | 124/24 |  |
| Adjusted HR (95%CI) ^a^ | 1 (Ref.) | 0.64 (0.38-1.10) | 0.38 (0.22-0.63 | 0.29 (0.18-0.48) | 0.28 (0.17-0.46) | 0.18 (0.10-0.32) | <0.001 |
| **Abdominal obesity** |  |  |  |  |  |  |  |
| *N* / deaths | 60/46 | 169/105 | 360/172 | 696/236 | 683/173 | 276/60 |  |
| Adjusted HR (95%CI) ^a^ | 1 (Ref.) | 0.63 (0.44-0.90) | 0.44 (0.31-0.62) | 0.34 (0.24-0.48) | 0.26 (0.18-0.36) | 0.22 (0.15-0.33) | <0.001 |
| **No hypertension** |  |  |  |  |  |  |  |
| *N* / deaths | 22/19 | 73/46 | 181/80 | 362/109 | 345/74 | 134/22 |  |
| Adjusted HR (95%CI) ^a^ | 1 (Ref.) | 0.43 (0.23-0.80) | 0.27 (0.15-0.49) | 0.17 (0.10-0.30) | 0.14 (0.08-0.25) | 0.09 (0.05-0.20) | <0.001 |
| **Hypertension** |  |  |  |  |  |  |  |
| *N* / deaths | 69/54 | 184/120 | 423/205 | 722/258 | 684/194 | 266/63 |  |
| Adjusted HR (95%CI) ^a^ | 1 (Ref.) | 0.65 (0.47-0.90) | 0.44 (0.32-0.60) | 0.36 (0.27-0.48) | 0.30 (0.22-0.41) | 0.24 (0.17-0.34) | <0.001 |
| **No hypercholesterolemia** |  |  |  |  |  |  |  |
| *N* / deaths | 79/63 | 191/126 | 454/224 | 813/299 | 763/211 | 292/65 |  |
| Adjusted HR (95%CI) ^a^ | 1 (Ref.) | 0.58 (0.41-0.82) | 0.41 (0.30-0.57) | 0.33 (0.24-0.45) | 0.27 (0.19-0.37) | 0.19 (0.13-0.27) | <0.001 |
| **Hypercholesterolemia** |  |  |  |  |  |  |  |
| *N* / deaths | 12/10 | 66/41 | 150/61 | 271/68 | 266/56 | 108/20 |  |
| Adjusted HR (95%CI) ^a^ | NA | 1 (Reference) ^b^ | 0.51 (0.34-0.77) | 0.39 (0.27-0.57) | 0.31 (0.20-0.46) | 0.35 (0.21-0.58) | <0.001 |
| **No comorbidity** |  |  |  |  |  |  |  |
| *N* / deaths | 58/46 | 166/104 | 444/189 | 839/263 | 801/191 | 327/68 |  |
| Adjusted HR (95%CI) ^a^ | 1 (Ref.) | 0.59 (0.40-0.88) | 0.37 (0.26-0.55) | 0.30 (0.31-0.44) | 0.24 (0.17-0.35) | 0.19 (0.13-0.29) | <0.001 |
| **≥ 1 comorbidities ^c^** |  |  |  |  |  |  |  |
| *N* / deaths | 33/28 | 91/62 | 160/96 | 245/104 | 228/76 | 73/17 |  |
| Adjusted HR (95%CI) ^a^ | 1 (Ref.) | 0.76 (0.47-1.22) | 0.52 (0.33-0.80) | 0.38 (0.25-0.57) | 0.34 (0.22-0.53) | 0.23 (0.13-0.40) | <0.001 |

HR: Hazard ratio; CI: Confidence interval; NA: not applicable. ^a^HRs are adjusted as in Model 2 in table 2. ^b^ Participants with 0-1 health behaviors were merged in this group. ^c^ Coronary heart disease, stroke, diabetes mellitus, hip fracture, and cancer at any site. Positive health behaviors: never smoking or quitting tobacco >15 years; being very/moderately physically active; having a healthy diet score ≥median in the cohort; sleeping 7-8 h/d; sitting time <8 h/d; interaction with friends daily.
